# Urban–Rural Disparities in Energy Intake and Contribution of Fat and Animal Source Foods in Chinese Children Aged 4–17 Years

**DOI:** 10.3390/nu9050526

**Published:** 2017-05-21

**Authors:** Ji Zhang, Dantong Wang, Alison L. Eldridge, Feifei Huang, Yifei Ouyang, Huijun Wang, Bing Zhang

**Affiliations:** 1 National Institute for Nutrition and Health, Chinese Center for Disease Prevention and Control, Beijing 100050, China; zhangji@chinacdc.cn or shion_zhang@163.com (J.Z); fayekobe@163.com (F.H.); hollyfaye@126.com (Y.O.); wanghj@chinacdc.cn (H.W.); 2 Nestlé Research Center, Lausanne 1000, Switzerland; dantong.wang@rdls.nestle.com (D.W); alison.eldridge@rdls.nestle.com (A.L.E.)

**Keywords:** dietary energy intake, fat, animal source foods, children and adolescents, urbanization, China

## Abstract

Objective: Excessive energy intake and poor food choices are major health concerns associated with overweight and obesity risk. This study aims to explore disparities in energy intake and the contributions from fat and animal source foods among Chinese school-aged children and adolescents in different communities based on urbanization levels. Design: Three consecutive 24 h recalls were used to assess dietary intake. Subjects’ height and weight were measured using standard equipment. Standardized questionnaires were used to collect household demographic and socioeconomic characteristics by trained interviewers. Setting: The 2011 China Health and Nutrition Survey is part of an ongoing longitudinal household survey across 228 communities in nine provinces and three mega-cities in China. Subjects consisted of children aged 4–17 years (*n* = 1866; 968 boys and 898 girls). Results: The estimated average energy intake was 1604 kcal/day (1706 kcal/day for boys and 1493 kcal/day for girls). Proportions of energy from fat and animal source foods were 36.8% and 19.8% respectively and did not differ by gender. Total energy intake showed no significant disparity, but the proportion of energy from fat and animal source foods increased with increasing urbanization levels and increasing household income level. The largest difference in consumption percentages between children in rural areas and those in highly urban areas was for milk and dairy products (14.8% versus 74.4%) and the smallest difference was seen in percent consuming meat and meat products (83.1% versus 97.1%). Conclusions: Results of this study highlight the need for developing and implementing community-specific strategies to improve Chinese children’s diet quality.

## 1. Introduction

Sufficient dietary energy intake is important for children and adolescents to support growth and development and to cover daily energy expenditure. On the other hand, excessive energy intake and poor food choices are major health concerns associated with risk for development of overweight and obesity [1,2,3] and obesity-related diseases such as diabetes [4,5] and hypertension [6,7]. With the emergence of the double burden of under- and over-nutrition in many countries, control of energy intake has become an important topic. In China, lifestyle and dietary patterns have changed concomitantly with rapid economic development and urbanization in the past 30 years [8,9], which has raised new health concerns such as overweight and obesity [10,11,12], but at the same time, in some less developed regions, the energy intake is still relatively low [13].

Changes are also occurring at the food source level. The consumption of a high fat diet, energy dense foods, and foods of animal origin have increased significantly in China [8,14,15]. Some animal source foods, such as meats, are higher in fat and provide more energy than plant source foods [16]. Among low-income countries, consumption of animal source foods has been associated with improved nutritional intakes [7,8,9,10,11,12,13,14,15,16,17,18,19], increased dietary diversity [19], and increased intake of protein, iron, and micronutrients [20,21]. In China, a two-year milk intervention at school improved growth and bone mineral accretion in girls [22]. There is concern, however, that excessive intake of animal source foods, especially meats, may increase the risk for cardiovascular disease [23] and that fat intake overall may increase the risk for obesity [24]. Therefore, the consumption of animal source foods should be sufficient to provide necessary nutrients and support growth but not so much as to increase the risk for non-communicable diseases.

In order to be better prepared to make dietary recommendations to manage energy intake in Chinese children, further research on dietary sources of energy, including fat and animal source foods is needed. Our objective is to evaluate the total energy intake and the percentages of energy from fat and animal source foods in a sample of 1866 Chinese children aged 4–17 years. Because of likely differences in food intake depending on community type [14], children from communities of different levels of urbanization (highly urban, moderately urban, and rural) are also compared.

## 2. Methods

### 2.1. Study Population

Data were obtained from the China Health and Nutrition Survey (CHNS) [25], an ongoing longitudinal survey started in 1989 that aims to assess the nutrition transition and its interaction with economic, sociological, and demographic factors in China. CHNS employs a multistage random cluster sampling process to draw households from urban and rural areas. The samples are taken from 228 communities in nine provinces from the northeast to southwest across China that represents a wide range of demographic differences and economic development stages. The survey has been conducted every two or three years. In 2011, three mega cities, Beijing, Shanghai, and Chongqing, were added in the survey. 

The final protocol of CHNS was approved by the Ethical Review Committee of Chinese Center for Disease Control and Preventive (No. 201524).

The current study used a cross-sectional sample drawn from subjects aged 4–17 years surveyed in the year of 2011. After excluding the extreme values of 38 children (lower than the 1st percentile, or higher than the 99th percentile of energy intake), 1866 children were included in the final analysis of energy intake level and its dietary sources. 

### 2.2. Estimation of Energy, Fat and Animal Source Foods

Dietary data were collected using three 24 h recalls. Trained investigators interviewed the participants in their homes on three consecutive days (two weekdays and one weekend day) to collect detailed information on the types and amounts of all foods and beverages consumed during the preceding 24 h. Photos and pictures were used to assist the quantification. Parents or caregivers provided the information on children younger than 12 years, but children aged 12 years and older responded on their own. Total energy and percentages of energy from fat and animal source foods were calculated based on the China Food Composition Table (FCT) [16]. Meat, poultry, eggs, fish and seafood, milk, and other dairy products were classified as animal source foods. For each food group, a child who consumed foods from that group at least once in three days was defined as a consumer, and the intake was calculated as the average of 3 days per consumer.

### 2.3. Community Types and Household Demographics

Children were surveyed at home in three mega cities and nine provinces in China. Mega cities included in this study were Beijing, Shanghai, and Chongqing, three big municipalities in China. We categorized communities into three types based on administrative classification that shares common socioeconomic features (such as population density, community infrastructure, accessibility to public transport, and hospitals and supermarkets): highly urban (city centers in mega cities), moderately urban (suburban areas of the mega cities and smaller cities), and rural areas. Family income was calculated according to standardized questionnaires. Individuals were ranked and divided into tertiles based on their household income per capita.

### 2.4. Child Characteristics

Children were divided into four groups by age: 4–6 years, 7–10 years, 11–13 years, and 14–17 years. Standard techniques and equipment were used to measure subjects’ height (without shoes) in meters and weight (in light clothing) in kilograms to calculate BMI (kilograms per square meter (kg/m^2^)). Children were defined as underweight, normal, overweight, and obese based on WHO BMI z-score cut-offs [26].

### 2.5. Statistical Methods

Means and standard errors (SE) of total energy intake, percentages of energy from fat and animal source foods were calculated in the total sample and by community types. Chi-square (*χ*^2^) and analysis of variance were used for univariate analysis, and Bonferroni adjustment was applied for multiple comparisons. SAS (version 9.3, SAS Institute Inc., Cary, NC, USA) was used in statistical analysis.

## 3. Results

Characteristics of the study population are presented in Table 1. Overall, approximately 70% of the children were of normal body weight, with about 20% overweight or obese and 10% underweight. Half of the children came from rural areas. Children in rural communities had lower rates of overweight and obesity, and lower household income levels.

### 3.1. Energy Intakes, Fat Intakes, and Animal Source Foods

Daily energy intake and percentages of energy from fat and animal source foods were stratified by gender, age group, weight status, family income level, and community type (Table 2). Total energy intake was significantly higher in boys compared to girls, and in older children compared to younger ones. No significant differences were detected in percentages of energy contribution from fat or animal source foods between boys and girls or among age groups. Underweight children were less likely to consume animal source foods, but no differences were observed in energy intake or percent of calories from fat by body weight status. Compared to children in lower-income families, children from higher-income families consumed significantly more energy with a higher proportion from fat and animal source foods. A similar relationship was observed in children living in highly urban communities, but with no significant difference in mean energy intake.

### 3.2. Impact of Community Type on Energy Intakes, Fat Intakes, and Animal Source Foods

Because of the differences seen by community type, we evaluated energy intake, fat intake, and animal source foods by level of urbanicity (Table 3). We found that children living in different communities had similar total energy intake, but the percentages of energy contributed by fat and animal source foods decreased significantly from highly urban communities to moderately urban and rural areas in a step-wise fashion. In highly urban communities, we saw a significantly higher consumption of fat and animal source foods in both boys and girls and across all ages. Children in highly urban households consumed the highest percentage of calories from fat, exceeding 40% of daily energy. Although the energy contribution from animal source foods was still maintained at the moderately urban level, a significantly higher total energy intake was found in the moderately urbanized areas in girls, 14–17-year-old teenagers, and children from higher income families. 

To gain insights on the energy contribution from various animal source foods, we further analyzed the consumption by food type (Table 4). Across all food types (meats, eggs, milk and dairy, poultry, and fish/seafood), the highest consumption occurred among children living in highly urban areas. The percentages of consumption all sub-groups of animal source foods decreased along with the decrease of urbanization levels, with the largest difference seen for the Milk and Milk Products group. Meat and meat products were the most frequently consumed animal source foods and contributed the most energy.

## 4. Discussion

Approximately half of the study population was living in rural areas in China, and nearly one fourth of the subjects were living in highly urbanized cities such as Beijing, Shanghai, and Chongqing. According to the China National Nutrition and Health Survey 2012 [27], the national prevalence of childhood overweight was 8.4% (same in urban and rural areas), and that of childhood obesity was 3.1% (3.3% in urban and 2.9% in rural areas). In this study, we found that the prevalence of overweight and obesity in school-aged children was 12.1% and 9.2%, respectively, both of which were much higher than the national prevalence. Higher obesity rates have been reported for children in Beijing (16.6% among school-aged children) [28] and Shanghai 19.8% in boys and 8.4 in girls) [29]. Therefore, the higher prevalence of overweight and obesity may be attributed to the oversampling of children living in mega-cities in this study. On the other hand, underweight children accounted for 10.2% of the whole study sample, similar to 9.0% reported in the 2012 National Survey [27], confirming the existence of the double burden of over- and under-nutrition in Chinese children [10]. Similarly, the phenomenon of the double burden has been also reported in other countries [30,31,32]. As expected, obesity and overweight were more common in urban areas, while underweight was mainly a problem in rural areas, indicating the need for regionally specific public policies focusing on obesity and underweight, respectively.

The results of China National Nutrition and Health Survey showed that, in 2002 [13], the average energy intake of Chinese boys aged 4–17 ranged from 1481 to 2346 kcal/day, and 1391 to 1991 kcal/day for girls. In the current study, we found lower mean total energy intakes in every age group with only 24.4% of children reaching the Estimated Energy Requirement (EER) for Chinese children (data not shown) [33]. However, this does not necessarily mean that children are suffering from energy deficiency. Self-reported dietary data suffers from measurement errors, and energy underreporting has been reported [34,35,36]. Our data on total energy intakes may also be under-estimated in this population, which suggests a higher proportion of children in fact have adequate dietary energy intakes. In other research, average energy underreporting on dietary recalls was 6–16% in young adults compared to doubly labeled water measurement, which is considered to be unbiased measurement of energy metabolism [35], but no such data are available for children and adolescents. Moreover, researchers pointed out that low energy reporters tend to report lower intakes of foods high in fats and sweets [34]. 

The energy contribution from fat was higher in the current study than that reported in the national survey in 2002 [27]. With an average percentage of 36.8% of energy from fat, this exceeds the recommended proportion of 30% [37] from the Chinese dietary guidelines. Even among children living in rural communities, the percentage of energy from fat at 33.6% still exceeded the recommended 30%. Researchers have been reporting a trend of decreasing daily energy intakes and rising fat energy contribution, or increasing consumption of energy dense foods, in China [12]. Considering the adverse health effects of excessive animal source fat and total fat intake [23,24,38], the higher percentage of energy from fat is worrisome and should be modified.

In line with previous findings, the percentage of energy from animal source foods also indicates a transition in dietary patterns, with a rise from 12% in 2002 [15] up to 19% now in Chinese children. In our study, more than 80% of the children consumed meat and meat products at least once in the three days of dietary recalls in all three community types. However, the consumption of milk and milk products differed substantially with more than 70% of the children in highly urban communities, which was almost double the percentage of moderately urban communities (39%) and five times that of rural communities (15%). Different animal source foods provide different nutrients in the diet, so separate recommendations for each of the different types of animal source foods are given. In the new Chinese Dietary Guidance (2016) [37], the Chinese Nutrition Society encourages people to consume 300 g of milk or milk products on a daily basis. Results from this study showed that it is challenging to meet this recommendation, particularly in less urbanized communities.

Energy provided by animal source foods ranged from 16.5 to 27.3% with rising urbanization levels. Socioeconomic status (SES) is frequently reported to be associated with health behaviors and health outcomes [31,39,40], including in China [12,41]. A systematic review [39] showed that high SES or living in urban areas is associated with overall healthier dietary patterns, but these factors are also related to higher total energy and fat intakes in low- and middle-income countries, including China. In the current study, we found that the impact of SES on total energy intake and fat contribution was significant in rural communities, but not in highly urban communities. Moderately urban communities were between the two, showing a transition. These data confirm that the impact of SES on dietary energy intake is associated with the urbanization process.

Given the result that the total energy intake as well as contribution of fat is either equal or even higher in underweight children as compared to obese children in some subgroups, further research that focus on the contribution of carbohydrates and proteins are needed to explore the optimal proportions of the three macronutrients.

One limitation of this study is that the dietary intake data was self-reported, calculated based on three 24 h recalls. Concern about the reliability of self-reported dietary data has been raised among the scientific community [34,42], and our study also found low reported energy intakes in many Chinese children. Future studies should aim to improve collection of energy intake data by incorporating objective methods in at least a subsample of the whole population to adjust for the measurement error in reported energy intakes. Another limitation is that this is a cross-sectional study, and no causal relationships can be drawn.

## 5. Conclusions

In conclusion, although the reported energy intake was similar, we found significant differences by community type in the energy contribution from fat and animal source foods in Chinese children. The proportion of energy from fat and animal source foods increased along with increasing urbanization, as did the consumption of different types of animal source foods. The impact of family income showed differences in different community types. The results of this study highlight the need to promote dietary guidance by developing and implementing community-specific strategies to improve the quality of Chinese children’s diets.

## Figures and Tables

**Table 1:** Characteristics of surveyed children by community types, CHNS 2011.

	Total		Highly Urban		Moderately Urban		Rural	
	*n*	%	*n*	%	*n*	%	*n*	%
Total	1866	100.0	416	22.3	503	27.0	947	50.8
Gender								
Boys	968	51.9	209	50.2	261	51.9	498	52.6
Girls	898	48.1	207	49.8	242	48.1	449	47.4
Age								
4–6 years	448	24.0	95	22.8	127	25.3	226	23.9
7–10 years	613	32.9	131	31.5	149	29.6	333	35.2
11–13 years	398	21.3	87	20.9	102	20.3	209	22.1
14–17 years	407	21.8	103	24.8	125	24.9	179	18.9
Body Weight								
Underweight	190	10.2	12	2.9	62	12.3	116	12.2
Normal	1278	68.5	277	66.6	322	64.0	679	71.7
Overweight	226	12.1	72	17.3	67	13.3	87	9.2
Obese	172	9.2	55	13.2	52	10.3	65	6.9

**Table 2:** Daily energy intake and energy sources from fat and animal source foods, CHNS 2011.

	Energy Intake (Kcal/day)		Fat Intake (% of Total Energy)		Animal Source Food Intake (% of Total Energy)	
	Mean	SE	Mean	SE	Mean	SE
Total	1604	14	36.8	0.3	19.8	0.3
Gender						
Boys (ref) ^1^	1706	20	36.3	0.4	19.7	0.4
Girls	1493 ^a^	18	37.3	0.4	19.9	0.4
Age						
4–6 years (ref) ^1^	1299	23	37.2	0.5	20.4	0.6
7–10 years	1529 ^a^	21	37.5	0.5	20.5	0.5
11–13 years	1746 ^a^	30	35.8	0.6	18.5	0.6
14–17 years	1911 ^a^	32	36.2	0.6	19.4	0.6
Body Weight						
Underweight	1598	43	35.2	0.9	16.3 ^a^	0.9
Normal (ref) ^1^	1593	17	37.0	0.3	20.0	0.3
Overweight	1634	39	36.4	0.8	20.5	0.8
Obese	1651	48	37.6	0.9	21.8	1.0
Household Income Per Capita						
Lower tertile (ref) ^1^	1530	24	34.3	0.5	15.8	0.5
Middle tertile	1566 ^a^	24	37.0 ^a^	0.5	19.7 ^a^	0.5
Higher tertile	1716 ^a,b^	25	38.9 ^a^	0.4	23.8 ^a,b^	0.5
Community type						
Highly urban (ref) ^1^	1602	27	41.6	0.5	27.3	0.6
Moderately urban	1651	29	38.7 ^a^	0.6	20.0 ^a^	0.5
Rural	1579	19	33.6 ^a,b^	0.4	16.5 ^a,b^	0.4

^1^ Reference group. ^a^ *p* < 0.05 when compared to the referent; ^b^ *p* < 0.05 when “Higher tertile” is compared to “Middle tertile” (for Household income per capita) or “Rural” is compared “Moderately urban” (for Community type).

**Table 3:** Daily energy intake, percent fat and percent of energy from animal source foods, by community types, CHNS2011.

	Energy (Kcal/day) ^1^						Fat (% of Daily Energy) ^1^						Animal Source Foods (% of Daily Energy) ^1^					
	Highly Urban		Moderately Urban		Rural		Highly Urban		Moderately Urban		Rural		Highly Urban		Moderately Urban		Rural	
	Mean	SE	Mean	SE	Mean	SE	Mean	SE	Mean	SE	Mean	SE	Mean	SE	Mean	SE	Mean	SE
Total	1602	27	1651	29	1579	19	41.6 ^b^	0.5	38.7 ^a^	0.6	33.7 ^a,b^	0.4	27.3 ^b^	0.6	20 ^a^	0.5	16.5 ^a,b^	0.4
Gender																		
Boys	1753	42	1720	41	1679	28	41.6 ^b^	0.7	38.2 ^a^	0.8	33.1 ^a,b^	0.5	27.9 ^b^	0.9	19.6 ^a^	0.7	16.4 ^a,b^	0.5
Girls	1449 ^b^	31	1578 ^a^	40	1468 ^b^	26	41.6	0.7	39.2	0.8	34.2 ^a,b^	0.6	26.6 ^b^	0.8	20.4 ^a^	0.8	16.6 ^a,b^	0.5
Age																		
4–6 years	1390	52	1281	46	1272	30	40.6	0.9	39.7	1.2	34.4 ^a,b^	0.7	26.4 ^b^	1.2	20.6 ^a^	1.1	17.9 ^a^	0.8
7–10 years	1539	45	1514	41	1532	30	43.2	1	40.2	1.1	34.1 ^a,b^	0.7	28.3 ^b^	1.1	21.3 ^a^	1	17 ^a,b^	0.6
11–13 years	1710	58	1855	65	1709	41	41.5 ^b^	1.1	37 ^a^	1.2	32.8 ^a,b^	0.8	27.1 ^b^	1.4	18.7 ^a^	1.1	14.9 ^a,b^	0.7
14–17 years	1786 ^b^	59	2026 ^a^	60	1903	47	40.6	1	37.3	1.1	32.8 ^a,b^	0.8	26.9 ^b^	1	18.8 ^a^	1	15.6 ^a,b^	0.8
Body Weight																		
Underweight	1648	125	1495	84	1648	53	41.3	2.5	35.8	1.6	34.3	1.1	20	2.2	17.2	1.7	15.5	1.2
Normal	1595	34	1684	36	1549 ^b^	23	42.1 ^b^	0.6	39.4 ^a^	0.7	33.8 ^a,b^	0.4	27.5 ^b^	0.7	20.4 ^a^	0.6	16.7 ^a,b^	0.4
Overweight	1588	63	1590	77	1706	64	40	1.2	37.1	1.6	32.8 ^a^	1.1	26.4 ^b^	1.3	18.7 ^a^	1.4	16.8 ^a^	1.1
Obese	1646	81	1718	96	1602	77	41.4	1.4	40.1	1.6	32.5 ^a,b^	1.3	28.7 ^b^	1.8	22 ^a^	1.8	15.8 ^a,b^	1.2
Household income per capita																		
Lower tertile	1552	75	1514	52	1533	30	40.7 ^b^	1.6	38.1 ^a^	1.2	31.9 ^a,b^	0.6	23.1 ^b^	1.6	17.2 ^a^	1	14.2 ^a,b^	0.5
Middle tertile	1577	50	1560	45	1566	33	42.1 ^b^	1	37.8 ^a^	0.9	34.7 ^a,b^	0.6	26.7 ^b^	1.1	18.9 ^a^	0.8	17.5 ^a^	0.7
Higher tertile	1632 ^b^	37	1895 ^a^	54	1680 ^b^	41	41.5	0.6	39.8	0.9	35.4 ^a,b^	0.7	28.6 ^b^	0.8	23.7 ^a^	1	18.9 ^a,b^	0.6

**^1^** Statistical comparisons are across Community types within a category (energy, fat, animal source foods); ^a^ *p* < 0.05 when compared to “Highly Urban”; ^b^ *p* < 0.05 when compared to “Moderately Urban”.

**Table 4:** Energy contribution from different animal source foods among consumers and by community type, CHNS2011.

Animal Source Foods	Highly Urban ^1^					Moderately Urban ^1^					Rural ^1^				
	% Consuming	kcal/day		% Daily Energy		% Consuming	kcal/day		% Daily Energy		% Consuming	kcal/day		% Daily Energy	
		Mean	SE	Mean	SE		Mean	SE	Mean	SE		Mean	SE	Mean	SE
Meat and Meat Products	97.1	223	9	14.2 ^b^	0.5	92.3	200	8	13.4 ^a^	0.4	83.1	181 ^a^	5	13.7 ^a^	0.3
Eggs and Egg Products	84.4	65 ^b^	2	4.4 ^b^	0.2	73.6	51 ^a^	2	3.2 ^a^	0.1	62.2	50 ^a^	1	3.3 ^a^	0.1
Milk and Milk Products	70.4	120 ^b^	4	7.8 ^b^	0.3	38.6	116 ^a^	14	6.9 ^a^	0.6	14.8	99 ^a,b^	9	6.0 ^a,b^	0.4
Poultry and Poultry Products	60.3	74 ^b^	4	4.6 ^b^	0.3	37.4	72 ^a^	4	4.4 ^a^	0.3	31.1	72 ^a^	4	4.5 ^a^	0.2
Fish and Fish Products	51.9	40 ^b^	2	2.6 ^b^	0.1	40.4	38 ^a^	2	2.3 ^a^	0.2	36.6	33 ^a,b^	1	1.9 ^a,b^	0.1

**^1^** Statistical comparisons are across Community types within a food group; ^a^ *p* < 0.05 when compared to “Highly Urban”; ^b^ *p* < 0.05 when compared to “Moderately Urban”.
